# Associations between insomnia and large vessel occlusion acute ischemic stroke: An observational study

**DOI:** 10.1016/j.clinsp.2023.100297

**Published:** 2023-11-03

**Authors:** Huali Xu, Weili Li, Jiahao Chen, Piao Zhang, Siming Rong, Jinping Tian, Yuqian Zhang, Yanke Li, Zhenzhen Cui, Yuhu Zhang

**Affiliations:** a Department of Neurology, Guangdong Neuroscience Institute, Guangdong Provincial People's Hospital (Guangdong Academy of Medical Sciences), Southern Medical University, Guangzhou, Guangdong Province, China; b Department of Neurology, Binzhou Central Hospital, Binzhou Medical College, Binzhou, Shandong Province, China; c Cerebrovascular Diseases Research Institute, Xuanwu Hospital, Capital Medical University, Beijing, China; d Department of Neurobiology, Capital Medical University, Beijing, China; e Department of Information, Binzhou Central Hospital, Binzhou Medical College, Binzhou, Shandong Province, China

**Keywords:** Insomnia, Acute ischemic stroke, Endovascular treatment, Inflammation, Oxidative stress, Prognosis

## Abstract

**Objectives:**

This study explored the association between insomnia and the clinical outcome of large vessel occlusion Acute Ischemic Stroke (AIS) and attempted to explore its potential mechanisms from the perspectives of inflammation and oxidative stress.

**Methods:**

AIS patients who underwent endovascular treatment for large vessel occlusion at Binzhou Central Hospital from 2018 to 2022 (n = 508) were included. Patients were divided into an insomnia group and a non-insomnia group. Insomnia was judged by self-reported Athens Insomnia Scale score. Regression analysis was used to compare the differences in the 24-hour and 7-day National Institutes of Health Stroke Scale (NIHSS) score, Early Neurological Deterioration (END), early adverse event incidence, 90-day prognosis and mortality, and serum biomarkers levels.

**Results:**

The incidence of insomnia in the study population was 39.6% (n = 144, insomnia group; n = 364, non-insomnia group). Compared with the non-insomnia group, a worse prognosis outcome (63% vs. 49%, adjusted rate ratio: 1.8, 95% Confidence Interval: 1.2–3.7; p = 0.016), higher 24-h and 7-day NIHSS score (17 [9–36] vs. 13 [5–20]; p = 0.024, and 11 [4‒24) vs. 8 [2‒14]; p = 0.031, respectively), higher END (24% vs. 15%, p = 0.022), and higher incidence of adverse events were observed in the insomnia group (79% vs. 59%, p = 0.010). The 90-day mortality was higher in the insomnia group than that in the non-insomnia group (22% vs. 17%), however, such a difference was not statistically significant.

**Conclusion:**

Insomnia is closely related to the clinical outcome of AIS with large vessel occlusion, and inflammation and oxidative stress mechanisms may be involved.

## Highlights

• The incidence of insomnia is higher in the acute ischemic stroke population.
• Insomnia is closely related to the clinical outcome of acute ischemic stroke with large vessel occlusion.
• The effects of Insomnia on acute ischemic stroke may involve inflammation and oxidative stress mechanisms.

## Introduction

Sleeping well helps the body eliminate fatigue, protect the brain, and restore physical strength and energy levels; it is an important factor in maintaining the normal physiological functions of the body.^1^^,^^2^ However, in our society and with the rapid development of the economy, many people face insomnia due to various external pressures, affecting not only the middle-aged and the elderly but also the younger people.^3^^,^^4^ Insomnia is the most common sleep disorder.^5^ Insomnia pertains to the disorder of sleep quantity, quality, time, and rhythm.^6^ For a long time, extreme sleep duration has been considered an important factor leading to the risk of Cardio-cerebral vascular diseases.^7^^,^^8^ Therefore, more and more people are paying attention to insomnia problems and their effects on various health outcomes including Cardio-cerebral vascular diseases.^9^

Several studies have shown that insufficient sleep may increase the risk of stroke.^10^, ^11^, ^12^ Some researchers have also focused on the association between sleep and stroke,which seems to be different between men and women.^13^ The association between self-reported sleep duration and combined ischemic and hemorrhagic stroke mortality has been investigated in other cohort studies.^14^, ^15^, ^16^

However, there is no definite conclusion regarding the relationship between stroke and insomnia. In addition, only a few studies^17^^,^^18^ have reported the impact of insomnia on the outcome of stroke and the mechanisms by which insomnia affects the occurrence and development of stroke. More importantly, few studies have been conducted on the relationship between insomnia and stroke in the Chinese population, where the incidence of stroke has increased rapidly in the past decades.^19^

Endovascular Treatment (EVT) has been recommended as the first-line treatment for large vessel occlusion in Acute Ischemic Stroke (AIS). However, it is unclear whether insomnia affects the clinical outcome of EVT. This study aimed to investigate the effect of insomnia on the clinical outcome of AIS with large vessel occlusion and to analyze the mechanism of insomnia in AIS from the perspective of inflammation and oxidative stress. There are a large number of people with insomnia in the general population. If it can be confirmed that insomnia is an independent risk factor affecting the prognosis of AIS, early intervention of the insomnia population will greatly improve the clinical outcome of stroke, which has important clinical significance.

## Methods

### Patient selection

**Fig. 1:**
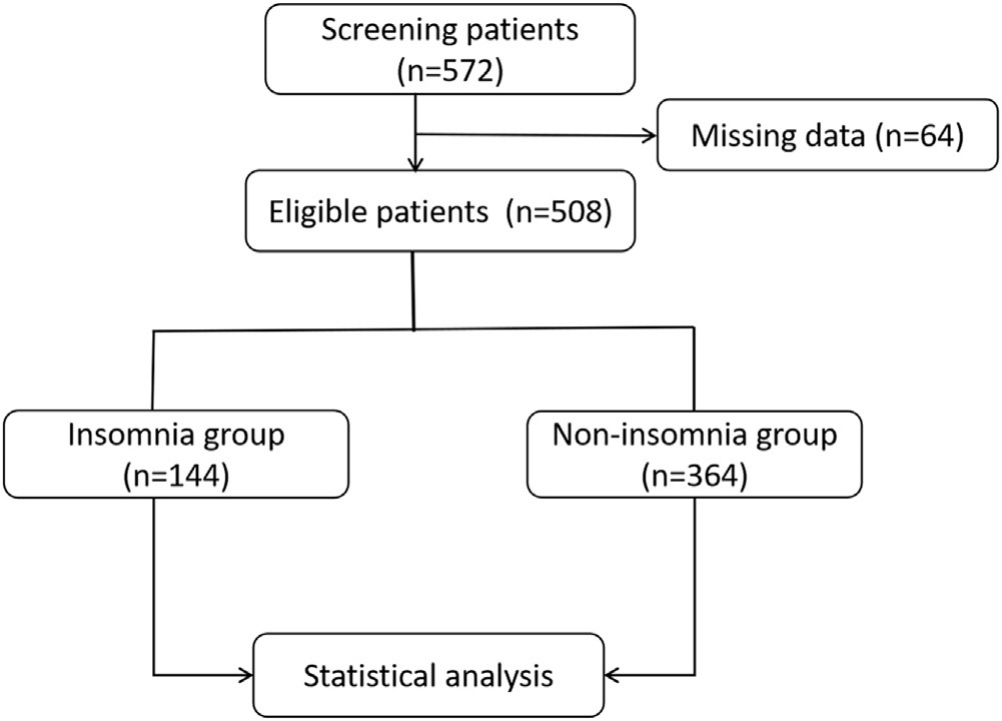
Flow chart of patient grouping. A total of 572 patients were screened for acute ischemic stroke with large vessel occlusion after endovascular treatment, and 64 patients with incomplete data were excluded; 508 patients were finally eligible, including 144 patients with insomnia and 364 patients without insomnia.

In this observational study, the authors analyzed patients with AIS who received Emergency Endovascular Treatment (EVT) in Binzhou Central Hospital from 2018 to 2022. The inclusion criteria were as follows: age ≥18 years, clinical symptoms, and imaging diagnosis consistent with AIS with large vessel occlusion, and the patients underwent EVT. Informed consent was obtained for the data analysis. The exclusion criteria included: life expectancy < 90 days, inability to complete the 90-day follow-up, and evidence of an intracranial tumor. The flowchart of the study is shown in Fig. 1.

### Study design

This was a single-center case-control study. This study was approved by the Ethics Committee of Binzhou Central Hospital. The participating patients with AIS who received EVT for large vessel occlusion were divided into two groups according to the self-reported questionnaire: the insomnia group and the non-insomnia group. All patients were measured by self-reported Athens Insomnia Scale score. The Athens Insomnia Scale consists of 8 questions that explore the perception of difficulty in inducing sleep, waking up during the night, waking up early in the morning, total sleep time, general quality of sleep, feeling of wellness, general functioning, and somnolence during the day. Each question on the Athens insomnia scale score is scored from 0‒3, where 0 means no problem and 3 means very serious problem. Athens insomnia scale score > 6 is defined as insomnia. Statistical analysis was used to compare the differences between the two groups in clinical prognosis (including the National Institutes of Health Stroke Scale [NIHSS] score at 24h and 7-days, and mRS score at 90 days), severe complications (including Early Neurological Deterioration [END], adverse event rate at 7-days, poor prognosis at 90 days and mortality at 90 days), and serum inflammation and oxidative stress biomarkers (including serum C-Reactive Protein [CRP], blood white blood cell count, blood neutrophil count, Hypoxia-Inducible Factor-1α [HIF-1α], and Malondialdehyde [MDA]), to explore the correlation between insomnia and AIS.

All patients had their follow-up via telephone or on-site by specially trained neurologists. The patients' sleep status, Athens Insomnia Scale score, and 90-day functional prognosis were obtained during the follow-ups.

### Assessment outcomes

The evaluation indicators included clinical prognosis, serious complications, and inflammatory indicators. The prognosis index included the 24-hour NIHSS score, changes in 24-hour NIHSS scores from baseline, 7-day NIHSS scores, and 90-day mRS scores. Severe complications included END, the incidence of adverse events within 1-week, a poor 90-day prognosis, and 90-day mortality. END is defined as an increase in the NIHSS score ≥4 points within 24–48 hours.^20^ Adverse events mainly included pulmonary infections, END, symptomatic intracranial hemorrhage, myocardial infarction, and deep vein thrombosis of the lower limb. Symptomatic intracranial hemorrhage was defined as intracranial hemorrhage confirmed by brain Computed Tomography (CT) or magnetic resonance image scanning within 22–36 hours, with an NIHSS score increase by ≥4 points according to the European Co-operative Acute Stroke Study-II (ECASS-II) diagnostic criteria. Intracranial hemorrhage was determined to be the main cause of neurological function deterioration.^21^ A poor outcome was defined as a modified Rankin scale (mRS) score of 3–6.^22^

All patients underwent EVT. Postoperative management was performed following perioperative management guidelines. As a senior stroke center, Binzhou Central Hospital can complete at least 100 EVT operations annually. Each surgeon has ample experience in ensuring operational stability.

According to the extended Thrombolysis In Cerebral Infarction (eTICI) grades standard, a grade ≥ 2b is defined as successful recanalization, and < 2b is defined as failed recanalization.^23^ The serum biomarkers included serum CRP, blood leukocyte counts, blood neutrophil counts, HIF-1α, and MDA. The above indicators were completed within 12 hours of admission. The measurement of inflammatory markers was routinely obtained through hospital biochemical laboratories, and oxidative stress markers (HIF-1α, and MDA) were measured using commercialized ELISA kits (Huaxingbio, China)

### Statistical analysis

Data are presented as mean ± standard deviation or median (Interquartile Range, IQR). For continuous variables, normal distribution was assessed using the Kolmogorov-Smirnov test. A Student's *t*-test or ANOVA variance test was conducted for normal/Gaussian distribution data. If the data did not exhibit a normal distribution, the Mann-Whitney test was used. For categorical variables, the authors used the chi-squared test or Fisher's exact test. To further eliminate the influence of confounding factors, the authors used multivariate regression analysis to explore the correlation between insomnia and stroke outcomes after adjusting for age, gender, onset to revascularization time, Alberta Stroke Program Early CT Score (ASPECTS), the Trial of Org 10172 in Acute Stroke Treatment (TOAST) classification, intravenous thrombolysis, and vascular occlusion location. All data were analyzed using SPSS software (version 26.0, SPSS Inc., USA); p < 0.05 was considered statistically significant.

## Results

### Baseline characteristics

From 2018 to 2022, the authors screened 572 patients diagnosed with AIS in the Binzhou Central Hospital who underwent EVT and excluded 64 patients with incomplete data. A total of 508 patients were included, 144 patients with insomnia and 364 patients without insomnia (Fig. 1). Insomnia accounted for 39.6% of the total study population. The baseline data of the two groups were statistically analyzed. The results showed statistical differences between the insomnia group and the non-insomnia group for age (64.7 ± 12.3 vs. 61.0 ± 10.3, p = 0.007); baseline NIHSS score (17 [13–33] vs. 14 [12–22], p = 0.016). There were no significant differences found in previous history (atrial fibrillation, hypertension, diabetes, and previous stroke history), ASPECTS, vascular occlusion location, TOAST classification, intravenous thrombolysis, laboratory indices (D-dimer, fibrinogen), time parameters (onset-to-door time, door-to-puncture time, puncture to reperfusion, stroke onset to reperfusion), and vascular recanalization rate between the two groups (p > 0.05) (Table 1).

**Table 1:** Characteristics of the patients at baseline.

Variable	Insomnia (n = 144)	Non-insomnia (n = 364)	p-value
Age ‒ year	64.7 ± 12.3	61.0 ± 10.3	0.025
Male sex ‒ n (%)	110 (76)	260 (71)	0.519
Atrial fibrillation ‒ n (%)	38 (26)	76 (21)	0.434
Diabetes mellitus ‒ n (%)	40 (28)	88 (24)	0.663
Hypertension ‒ n (%)	114 (75)	252 (69)	0.448
Previous ischemic stroke ‒ n (%)	60 (42)	140 (39)	0.572
NIHSS score (IQR)	17 (13‒33)	14 (12‒22)	0.016
ASPECTS on baseline CT (IQR)	9 (7‒10)	9 (8‒10)	0.254
Treatment with IVT ‒ n (%)	44 (31)	136 (37)	0.742
Occlusion site ‒ n (%)			
Anterior circulation	90 (63)	266 (73)	0.132
Posterior circulation	54 (37)	98 (27)	
Type of TOAST ‒ n (%)			
Cardiogenic embolism	100 (69)	230 (63)	0.426
Large-artery atherosclerosis	38 (26)	110 (30)	0.651
Others	6 (4)	24 (7)	0.657
Laboratory test			
D-Dimer (mmoL/L)	2.2 ± 1.2	2.8 ± 1.6	0.369
FIB (g/L)	3.4 ± 1.2	3.1 ± 0.8	0.140
Process measures ‒ min			
Onset-to-door time (IQR)	262 (177‒365)	287 (176‒360)	0.297
Door-to-puncture time (IQR)	98 (78‒136)	107 (87‒132)	0.225
Puncture to reperfusion (IQR)	77 (45‒131)	65 (39‒106)	0.482
Stroke onset to reperfusion (IQR)	415 (330‒515)	409 (310‒556)	0.788
eTICI Grade ≥2b ‒ n (%)	132 (92)	328 (90)	0.885

NIHSS, National Institutes of Health Stroke Scale; IQR, Interquartile Range; ASPECTS, Alberta Stroke Program Early CT Score; TOAST, Trial of Org 10172 in Acute Stroke Treatment; FIB, Fibrinogen; eTICI, Extended Thrombolysis In Cerebral Infarction.

### Athens Insomnia Scale Score analysis

The authors found that there were differences in the scale score for each item between the two groups (p < 0.001). In addition, the general quality of sleep had the highest score among these eight items, with 2.3 ± 0.5 in the insomnia group and 0.5 ± 0.5 in the non-insomnia group. The second highest score was total sleep time, with 2.2 ± 0.5 in the insomnia group and 0.4 ± 0.5 in the control group. This lowest score was in the general functioning item, with 1.3 ± 0.7 in the insomnia group and 0.1 ± 0.3 in the non-insomnia group (Table 2).

**Table 2:** Comparison of the athens insomnia scale scores.

Variable	Insomnia (n = 144)	Non-insomnia (n = 364)	p-value
Athens Insomnia Scale Score	13.8 ± 2.3	2.3± 2.1	p < 0.001
Each item of the Athens Insomnia Scale:			
Sleep	1.9 ± 0.5	0.4 ± 0.5	p < 0.001
Waking up during the night	1.4 ± 0.5	0.2 ± 0.4	p < 0.001
Waking up early in the morning	1.6 ± 0.5	0.4± 0.5	p < 0.001
Total sleep time	2.2 ±0.5	0.4 ± 0.5	p < 0.001
General quality of sleep	2.3 ± 0.5	0.5 ± 0.5	p < 0.001
Feeling of wellness	1.5 ± 0.7	0.1 ± 0.3	p < 0.001
General functioning	1.3 ± 0.7	0.1 ± 0.3	p < 0.001
Somnolence during the day	1.5 ± 0.5	0.2 ± 0.4	p < 0.001

Data are expressed as mean ± SD.

### Clinical prognosis analysis

**Fig. 2:**
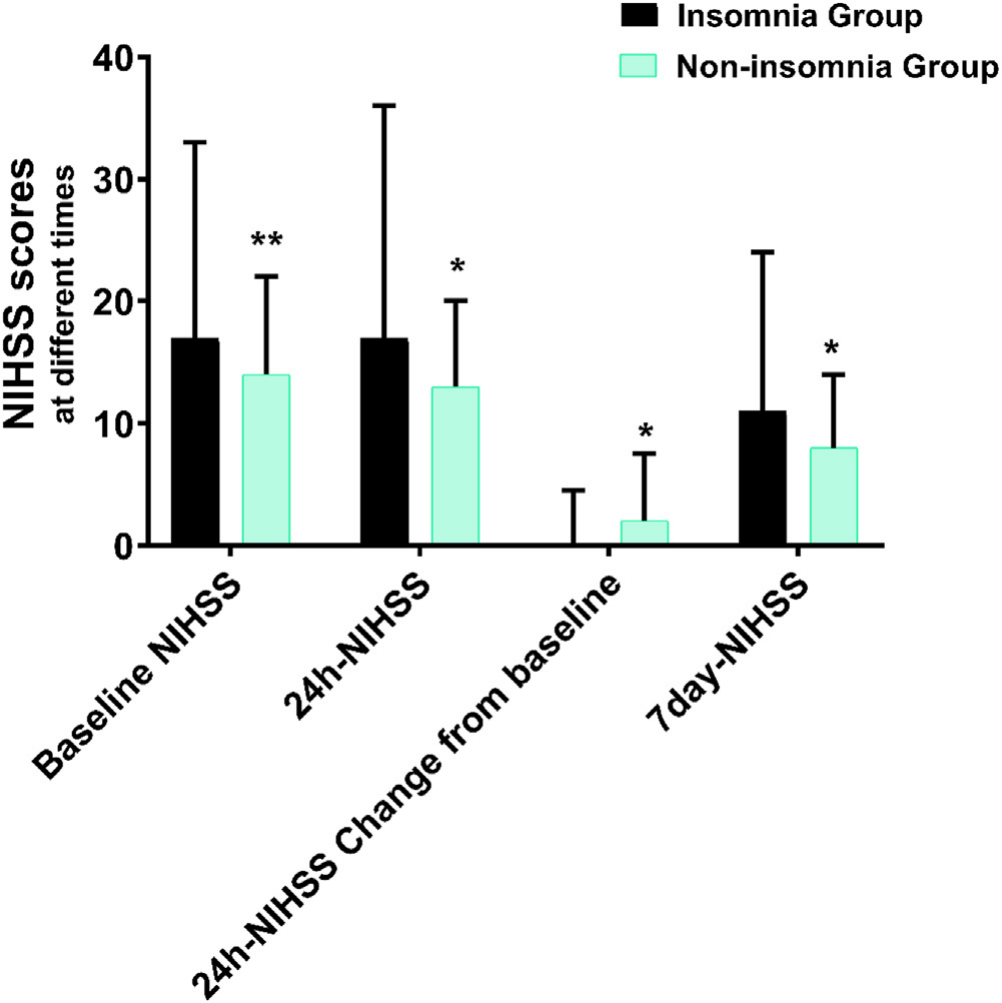
Comparison of NIHSS scores at different times. The NIHSS score at baseline [17 (13‒33) vs. 14 (12‒22)], 24-hour [17 (9‒36) vs. 13 (5‒20)], 24-hour changes from baseline [0 (-1‒4.5) vs. 2 (0‒7.5)] and 7-day NIHSS score [11 (4‒24) vs. 8 (2‒14)] were statistically significant in both groups (p < 0.05, respectively).

After adjusting for age, gender, onset to revascularization time, ASPECT score of admission, intravenous thrombolysis, TOAST classification, and vascular occlusion location, the authors found that the 24h-NIHSS score was independently associated with insomnia (17 [9–36] in the insomnia group vs. 13 [5–20] in the non-insomnia group, adjusted value -6.1, 95% CI -11.3 to -0.8, p = 0.024). In addition, for the change in 24h-NIHSS score from baseline (0 [-1‒4.5] in the insomnia group vs. 2 [0–7.5] in the non-insomnia group), 7 days NIHSS score (11 [4‒24] in the insomnia group vs. 8 [2‒14] in the non-insomnia group), and mRS score at 90 days (4 [2‒5] in the insomnia group vs. 3 [2‒5] in the non-insomnia group), there were significant differences between the two groups (adjusted value -3.7, 95% CI -6.7 to -0.6, p = 0.018; adjusted value -4.3, 95% CI -9.3 to -0.7, p = 0.031; adjusted value 1.8, 95% CI 1.2 to 3.7, p = 0.038), respectively, after adjusting for age, gender, onset to revascularization time, ASPECT score of admission, intravenous thrombolysis, TOAST classification, and vascular occlusion location (Table 3, Fig. 2).

**Table 3:** Comparison of study endpoints.

Outcomes	Insomnia (n = 144)	Non-insomnia (n = 364)	Measure of effect	Adjusted value^a^ (95% CI)	p-value
**Clinical prognosis**					
24-hour NIHSS score	17 (9‒36)	13 (5‒20)	Mean difference	-6.1(-11.3‒0.8)	0.024
Change in NIHSS from baseline to 24h	0 (-1‒4.5)	2 (0‒7.5)	Mean difference	-3.7 (-6.7‒0.6)	0.018
7-day NIHSS score	11 (4‒24)	8 (2‒14)	Mean difference	-4.3 (‒9.3‒0.7)	0.031
90-day mRS score	4 (2‒5)	3 (2‒5)	Mean difference	1.8 (1.2–3.7)	0.038
**Risk of serious complications**					
Early neurological deterioration	34 (24)	56 (15)	Rate ratio	2.7 (1.2‒6.3)	0.022
Adverse events within 7-days	114 (79)	214 (59)	Rate ratio	2.6 (1.3‒5.3)	0.010
Poor prognosis (mRS: 3‒6) ‒ n (%)	90 (63)	178 (49)	Rate ratio	0.7 (0.5‒0.9)	0.016
Death at 90-days	32 (22)	61 (17)	Rate ratio	0.6 (0.2‒2.3)	0.191
**Inflammatory factors**					
C-reactive protein (mg/L)	23.5 ± 13.8	11.3 ± 5.6	Mean difference	17.2 (3.4‒40.0)	0.015
White blood cell count (10^9^ /L)	11.8 ± 4.0	9.1 ± 2.0	Mean difference	2.4 (1.3‒3.5)	p < 0.001
Neutrophil count (10^9^ /L)	10.4 ± 4.0	7.8 ± 2.1	Mean difference	2.2 (1.1‒3.3)	p < 0.001
**Oxidative stress biomakers**					
HIF-1α (ng/mL)	1100.1 ± 149.8	990.3 ± 131.0	Mean difference	3.6 (1.5‒6.5)	p < 0.001
MDA (ng/mL)	1.18 ± 0.11	0.81 ± 0.10	Mean difference	2.5 (1.3‒4.7)	0.003

NIHSS, National Institutes of Health Stroke Scale; mRS, modified Rankin scale; HIF-1α, hypoxia-inducible factor-1α; MDA, Malondialdehyde.

a Adjusted for age, gender, onset to revascularization time, Alberta Stroke Program Early CT Score (ASPECTS), the Trial of Org 10172 in Acute Stroke Treatment (TOAST) classification, intravenous thrombolysis, and vascular occlusion location.

### Complication risk assessment

**Fig. 3:**
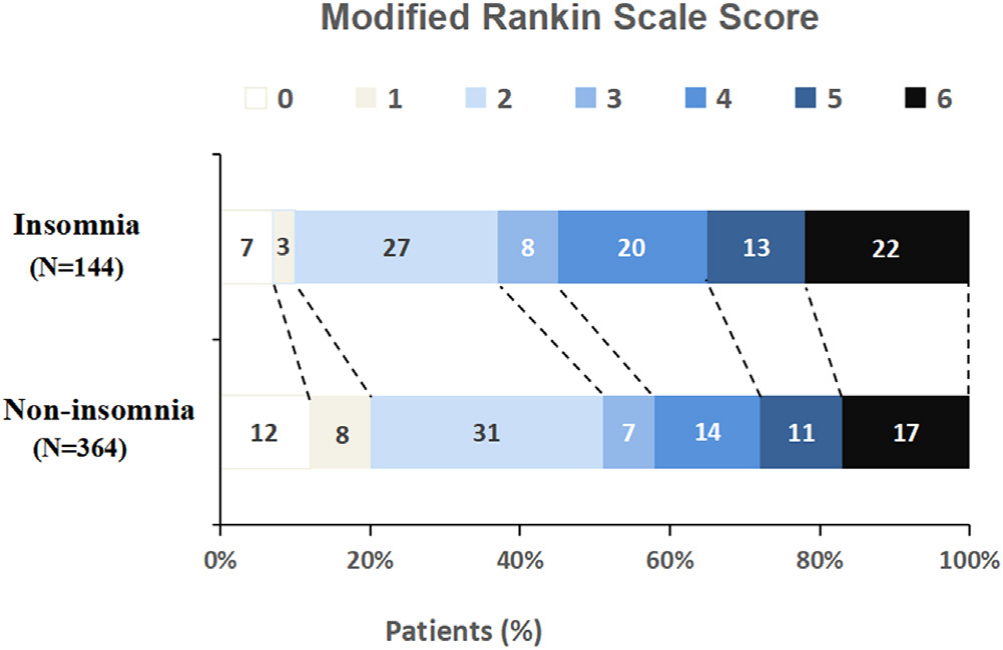
The distribution ratio of mRS Scores at 90-days. The poor prognosis (mRS3-6) in the insomnia group was significantly higher than that in the non-insomnia group (63% vs. 49%, adjusted value 0.7, 95% CI 0.5 to 0.9, p = 0.016).

The authors found that the incidence of END was 24% in the insomnia group and 15% in the non-insomnia group. The difference between the two groups was statistically significant (adjusted value 2.7, 95% CI 1.2 to 6.3, p = 0.022). Similarly, the incidence of adverse events and poor prognosis in the insomnia group was significantly higher than that in the non-insomnia group (79% vs. 59%, adjusted value 2.6, 95% CI 1.3 to 5.3, p = 0.01; 63% vs. 49%, adjusted value 0.7, 95% CI 0.5 to 0.9, p = 0.016) (Table 3, Fig. 3). Although the 90-day-mortality was higher in the insomnia group than that in the non-insomnia group (22% vs. 17%), there was no statistical difference between the two groups (p = 0.191) (Table 3).

### Analysis of serum biomarkers

To explore the relationship between inflammatory and oxidative stress mechanisms and insomnia, the authors analyzed the differences in blood leukocyte count, blood neutrophil count, serum CRP, HIF-1α, and MDA levels between the two groups. After adjusting for the abovementioned confounding factors, the authors found the following for the insomnia group vs. the non-insomnia group: serum CRP levels (23.5 ± 13.8 mg/L vs. 11.3 ± 5.6 mg/L, adjusted value 17.2, 95% CI 3.4 to 40.0, p = 0.015), white blood cell count (11.8 ± 4.0 vs. 9.1 ± 2.0, respectively, adjusted value 2.4, 95% CI 1.3 to 3.5, p < 0.001), neutrophil count (10.4 ± 4.0 vs. 7.8 ± 2.1, adjusted value 2.2, 95% CI 1.1 to 3.3, p < 0.001), HIF-1α (1100.1 ± 149.8 ng/mL vs. 990.3 ± 131.0 ng/mL, adjusted value 3.6, 95% CI 1.4 to 6.5, p < 0.001) and MDA (1.18 ± 0.11 vs. 0.81 ± 0.10 ng/mL, adjusted value 2.5, 95% CI 1.3 to 4.7, p = 0.003). These were all significantly higher in the insomnia group than in the non-insomnia group (Table 3).

## Discussion

This study explored the relationship between insomnia and AIS from the perspectives of clinical prognosis, inflammation, and oxidative stress. The results showed that the 24-hour NIHSS score, change in 24-hour NIHSS score from baseline, 7-day-NIHSS score, 90-day mRS score, END, poor prognosis (mRS:3-6), and incidence of adverse events were significantly higher in patients with insomnia. In addition, the levels of serum inflammatory factors and oxidative stress protein biomarkers in patients with insomnia were significantly higher than those in patients without insomnia. Although the 90-day mortality rate was higher in the insomnia group than in the non-insomnia group, such a difference was not statistically significant. It was worth noting that there were differences in the Athens Insomnia Scale Score for each item between the two groups, and general quality of sleep got the highest score among these eight items.

The novelty of this study lies in the following: it not only discussed the correlation between insomnia and the clinical outcome of AIS but the relationship between insomnia and AIS was also investigated based on inflammatory and oxidative stress. Furthermore, the study population included patients with AIS who had undergone EVT. This was not the case in previous studies. EVT for AIS is a hot topic in current research. Therefore, this study attempted to explore the correlation between insomnia and clinical outcomes in stroke patients after EVT.

It has been reported that sleep apnea severity was associated with END in AIS.^17^ In the same way, the present study found that insomnia was not only closely related to END but also to the incidence of early adverse events and poor prognosis at 90 days.

A study conducted in 2020 reported that sleep-disordered breathing is associated with impaired functional and cognitive function 90 days post-stroke and was not associated with neurologic outcomes (NIHSS) at 90 days.^18^ In contrast, the present study found that the insomnia group was significantly associated with early neurological dysfunction (24 hours and 7 days NIHSS score). For the 90-day functional results, the mRS scores in the insomnia group tended to be higher than those of the control group. This suggests that insomnia is independently associated with poor prognosis in AIS.

A recent prospective cohort study from China showed that short sleep duration was associated with the incidence of cardio-cerebral vascular disease.^24^ This is consistent with the present study showing an association between short sleep and stroke.

In addition, a recent cohort study from the United States in 2022 demonstrated significant associations between insomnia symptoms and all-cause mortality.^25^ In contrast, another Japanese cohort study found no association between sleep duration and stroke mortality in men but a trend in women.^26^ The present study confirmed that the 90-day mortality in the insomnia group increased by 5 percentage points compared to the control group, although there was no statistical difference. The apparent differences in results may be caused by differences in study design, as this study was retrospective, with confounding factors and a small sample size.

It is currently unclear how insomnia affects the occurrence and development of stroke. Sleep deprivation can trigger a cascade of inflammatory responses.^27^ In the central nervous system, sleep deprivation also induces peripheral blood cells to produce pro-inflammatory cytokines,^28^ which can act on peripheral macrophages to activate NF-KB or Toll-like receptors, and enhance the release of TNF-α, HMGB, IL-6, and other pro-inflammatory cytokines, leading to nerve damage.^29^^,^^30^ The present study also found that the leukocyte count, neutrophil count, and serum CRP levels in the peripheral blood of the insomnia group in the acute phase were higher than those in the non-insomnia group. This indicates that the inflammatory response of patients with insomnia is more obvious after a stroke. This may also explain why stroke patients in the insomnia group have poorer clinical outcomes, as there may be a close relationship with inflammatory mechanisms.

Inflammation and oxidative stress are usually concomitant, and scholars believe that one of the functions of sleep is to promote anti-oxidative mechanisms. This may be an adaptive response to sleep loss/deprivation as sleep disorders or sleep loss might induce oxidative stress.^31^ Previous studies have shown that sleep promotes the removal of free radicals accumulated during wakefulness.^32^ Hopps et al. detected significantly higher levels of MDA in patients with severe OSA compared with those who had mild and moderate disease severity.^33^ In addition, it is currently known that Reactive Oxygen Species (ROS) are potential inducers of the hypoxia-induced transcription Factor-1 (HIF-1).^34^ For instance, as one of the types of sleep disorder, Obstructive Sleep Apnea (OSA) could increase ROS/HIF-1α 'related oxidative stress and inflammation.^35^ It is not clear whether insomnia, like OSA, causes ROS/HIF-1α pathway activation, increasing oxidative stress and inflammation. This study confirmed that the serum levels of HIF-1α in patients with insomnia were significantly higher than those in non-insomnia patients, indicating that insomnia increased the risk of oxidative stress in AIS. This needs to be further confirmed through animal experiments.

### Limitations

This study had some limitations. Firstly, it was a retrospective study. The influence of confounding factors could not be balanced, even though the authors performed a regression analysis. Secondly, the sample size was small and it was just a case-control study. Therefore, a large-sample cohort study may be needed in the future. Thirdly, this study was unable to determine the association between each item of the Athens Insomnia Scale and poor stroke prognosis. It only answered the relationship between insomnia and stroke prognosis, even though the authors reported the scores of each Athens Insomnia Scale item in both groups. It is true that an individualized analysis for each item of the scale would allow a better correlation analysis. Finally, it was an observational study, which only observed the difference in inflammatory factors and oxidative stress protein between the two groups at the serum level. It could not determine the causal relationship between insomnia and inflammatory and oxidative stress mechanisms. Future interventional animal experiments are needed to explore whether insomnia directly or indirectly participates in the inflammatory and oxidative stress mechanism after AIS on the molecular and cellular levels. The authors need to search for more specific upstream key molecules or target proteins related to insomnia and AIS.

## Conclusions

Insomnia may be an important factor affecting the prognosis of patients with AIS, as it can increase early neurological impairment and other adverse events, and ultimately lead to poor prognosis. Inflammatory and oxidative stress mechanisms might also be involved in this process. In the future, large-scale prospective cohort studies and intervention-based experiments are needed to further verify the relationship between insomnia and AIS, and the probable underlying mechanisms.

## Conflicts of interest

The authors declare no conflicts of interest.

## References

[1] Matricciani L, Bin YS, Lallukka T, Kronholm E, Dumuid D, Paquet C (2017). Past, present, and future: trends in sleep duration and implications for public health. Sleep Health.

[2] Irwin MR. (2015). Why sleep is important for health: a psychoneuroimmunology perspective. Annu Rev Psychol.

[3] Kansagra S. (2020). Insomnia in Adolescents. Pediatrics.

[4] Yaremchuk K. (2018). Insomnia in the Elderly. Clin Geriatr Med.

[5] Korostovtseva L, Bochkarev M, Sviryaev Y. (2021). Sleep and Cardiovascular Risk. Sleep Med Clin.

[6] Sateia MJ. (2014). International classification of insomnia-third edition: highlights and modifications. Chest.

[7] Palma JA, Urrestarazu E, Iriarte J. (2013). Sleep loss as risk factor for neurologic disorders: a review. Sleep Med.

[8] Aldabal L, Bahammam AS. (2011). Metabolic, endocrine, and immune consequences of sleep deprivation. Open Respir Med J.

[9] Liu Q, Zhao J, Wang S. (2022). From cerebrovascular diseases to neuro-co-cardiological diseases: a challenge in the new epoch. Sci Bull (Beijing).

[10] Chaput JP. (2014). Sleep patterns, diet quality and energy balance. Physiol Behav.

[11] Grandner MA, Drummond SP. (2007). Who are the long sleepers? Towards an understanding of the mortality relationship. Sleep Med Rev.

[12] Helbig AK, Stöckl D, Heier M, Ladwig KH, Meisinger C. (2015). Symptoms of Insomnia and Sleep Duration and Their Association with Incident Strokes: Findings from the Population-Based MONICA/KORA Augsburg Cohort Study. PLoS One.

[13] Busch MA, Schienkiewitz A, Nowossadeck E, Gößwald A. (2013). Prevalence of stroke in adults aged 40 to 79 years in Germany: results of the German Health Interview and Examination Survey for Adults (DEGS1). Bundesgesundheitsblatt Gesundheitsforschung Gesundheitsschutz.

[14] Kim Y, Wilkens LR, Schembre SM, Henderson BE, Kolonel LN, Goodman MT (2013). Insufficient and excessive amounts of sleep increase the risk of premature death from cardiovascular and other diseases: the multiethnic cohort study. PrevMed.

[15] Amagai Y, Ishikawa S, Gotoh T, Doi Y, Kayaba K, Nakamura Y (2004). Sleep duration and mortality in Japan: the Jichi Medical School Cohort Study. J Epidemiol.

[16] Kakizaki M, Kuriyama S, Nakaya N, Sone T, Nagai M, Sugawara Y (2013). Long sleep duration and cause-specific mortality according to physical function and self-rated health: the Ohsaki Cohort Study. J Sleep Res.

[17] Yoon CW, Park H-K, Bae E-K, Rha J-H. (2020). Sleep Apnea and Early Neurological Deterioration in Acute Ischemic Stroke. J Stroke Cerebrovasc Dis.

[18] Lisabeth LD, Sánchez BN, Lim D, Chervin RD, Case E, Morgenstern LB (2019). Sleep-disordered breathing and poststroke outcomes. Ann Neurol.

[19] Liu S, Li Y, Zeng X, Wang H, Yin P, Wang L (2019). Burden of Cardiovascular Diseases in China, 1990-2016: Findings From the 2016 Global Burden of Disease Study. JAMA Cardiol.

[20] Hacke W, Kaste M, Bluhmki E, Brozman M, Dávalos A, Guidetti D (2008). Thrombolysis with alteplase 3 to 4.5 hours after acute ischemic stroke. N Engl J Med.

[21] Hacke W, Kaste M, Fieschi C, von Kummer R, Davalos A, Meier D (1998). Randomised double-blind placebo-controlled trial of thrombolytic therapy with intravenous alteplase in acute ischaemic stroke (ECASS II). Second European-Australasian Acute Stroke Study Investigators. Lancet.

[22] Albers GW, Marks MP, Kemp S, Christensen S, Tsai JP, Ortega-Gutierrez S (2018). Thrombectomy for Stroke at 6 to 16 Hours with Selection by Perfusion Imaging. N Engl J Med.

[23] Goyal M, Fargen KM, Turk AS, Mocco J, Liebeskind DS, Frei D (2014). 2C or not 2C: defining an improved revascularization grading scale and the need for standardization of angiography outcomes in stroke trials. J Neurointerv Surg.

[24] Ke J, Liu X, Ruan X, Wu K, Qiu H, Wang X, Li Z, Lin T. (2023). Short sleep duration associated with the incidence of cardio-cerebral vascular disease: a prospective cohort study in Shanghai, China. BMC Cardiovasc Disord.

[25] Mahmood A, Ray M, Ward KD, Dobalian A, Ahn S. (2022). Longitudinal associations between insomnia symptoms and all-cause mortality among middle-aged and older adults: a population-based cohort study. Sleep.

[26] Amagai Y, Ishikawa S, Gotoh T, Doi Y, Kayaba K, Nakamura Y (2004). Sleep duration and mortality in Japan: the Jichi Medical School Cohort Study. J Epidemiol.

[27] Xue R, Wan Y, Sun X, Zhang X, Gao W, Wu W (2019). Nicotinic Mitigation of Neuroinflammation and Oxidative Stress After Chronic Sleep Deprivation. Front Immunol.

[28] Chennaoui M, Gomez-Merino D, Drogou C, Geoffroy H, Dispersyn G, Langrume C (2015). Effects of exercise on brain and peripheral inflammatory biomarkers induced by total sleep deprivation in rats. J Inflamm (Lond).

[29] Bellesi M, de Vivo L, Chini M, Gilli F, Tononi G, Cirelli C (2017). Sleep Loss promotes astrocytic phagocytosis and microglial activation in mouse cerebral cortex. J Neurosci.

[30] Terrando N, Eriksson LI, Kyu Ryu J, Yang T, Monaco C, Feldmann M (2011). Resolving postoperative neuroinflammation and cognitive decline. Ann Neurol.

[31] Reimund E. (1994). The free radical flux theory of sleep. Med Hypotheses.

[32] Stanek A, Brożyna-Tkaczyk K, Myśliński W. (2021). Oxidative Stress Markers among Obstructive Sleep Apnea Patients. Oxid Med Cell Longev.

[33] Hopps E, Canino B, Calandrino V, Montana M, Lo Presti R, Caimi G (2014). Lipid peroxidation and protein oxidation are related to the severity of OSAS. Eur Rev Med Pharmacol Sci.

[34] Belaidi E, Morand J, Gras E, Pépin JL, Godin-Ribuot D. (2016). Targeting the ROS-HIF-1-endothelin axis as a therapeutic approach for the treatment of obstructive sleep apnea-related cardiovascular complications. Pharmacol Ther.

[35] Xiong M, Zhao Y, Mo H, Yang H, Yue F, Hu K. (2021). Intermittent hypoxia increases ROS/HIF-1α 'related oxidative stress and inflammation and worsens bleomycin-induced pulmonary fibrosis in adult male C57BL/6J mice. Int Immunopharmacol.

